# Enhanced Antiobesity Efficacy of Tryptophan Using the Nanoformulation of *Dendropanax morbifera* Extract Mediated with ZnO Nanoparticle

**DOI:** 10.3390/ma14040824

**Published:** 2021-02-09

**Authors:** Wenying You, Jong Chan Ahn, Vinothini Boopathi, Lakshminarayanan Arunkumar, Esrat Jahan Rupa, Reshmi Akter, Byoung Man Kong, Geun Sik Lee, Deok Chun Yang, Se Chan Kang, Jingjing Liu

**Affiliations:** 1 Weifang Engineering Vocational College, Qingzhou 262500, China; ywy0536@163.com; 2 Graduate School of Biotechnology, College of Life Sciences, Kyung Hee University, Yongin si, Gyeonggi 17104, Korea; jongchanahn@gmail.com (J.C.A.); vinothiniboopathi@khu.ac.kr (V.B.); arunmsc80@khu.ac.kr (L.A.); eshratrupa91@gmail.com (E.J.R.); Reshmiakterbph57@gmail.com (R.A.); kong2167@naver.com (B.M.K.); 3 Southwest Coast Hwangchil Cooperative, Chonnam National University, Yongbong-dong, Gwangju si 61186 and Yeji Bio, #411, Korea; 1357oklee@naver.com; 4 Jungwon University Industry Academic Cooperation Building, Goesan-gun, Chungbuk 28024, Korea

**Keywords:** *Dendropanax morbifera*, zinc nanoparticle, nanoemulsion, tryptophan, obesity 3T3-L1, RAW 264.7 cell lines

## Abstract

Green synthesis of metal nanoparticles from medicinal plants has provided a broad scope in biomedical research and functional food formulations due to low toxicity. *Dendropanax morbifera* (DM) is a versatile traditional medicine used for various inflammatory diseases due to its extensive antioxidant activity. We investigated DM as a natural capping agent for Zn^2+^ ions and coloaded it with tryptophan for its penetration and antiobesity behavior. DM zinc oxide nanoparticles (DM-ZnO NPs) were prepared and then entrapped with tryptophan (DM-ZnO-Try nanoemulsion (NE)) for stable formulation using the O/W nanoemulsion method. The hydrodynamic sizes measured by dynamic light scattering for DM-ZnO NPs and DM-ZnO-Try NE are about 146.26 ± 3.31 and 151.16 ± 3.59 nm, respectively. TEM and SEM reveal its morphology. In vitro analysis on both NPs and NE was non-toxic to RAW 264.7 and 3T3-L1 preadipocyte cell line. It significantly reduced the accumulated lipids through lipolysis performed at 10 ug/mL in 3T3-L1 preadipocyte cells. NE suppresses the differentiation of 3T3-L1 adipocytes and lowers triglycerides. Further, the substantial reduction of lipid content is evident with Oil Red O staining and OD measurement. In this present study, the synergetic effect of DM-ZnO NPs and tryptophan is reported, which provides a way for more detailed research on its efficacy for obesity and obesity-associated disorders.

## 1. Introduction

Recent developments and unique advancements in nanoscience and nanomaterials have established a novel platform for nanodrug delivery. This significant approach provides nanomaterials with enhanced stability, solubility, and bioefficiency under metabolic conditions [1]. Due to its effective advantages and target specificity at lower doses, this nanodrug carrier system is used for investigating various natural components like phenolic compounds, triterpenoids, and polyacetylene substances [2]. Polymeric conjugates, polymeric nanoparticles, and polymeric micelles use metal and non-metallic nanoparticles for drug loading by encapsulation or chemical conjugation and it is to get better biocompatibility, immunogenicity, stability, and low leakage of drugs for an effective drug delivery system [3]. Though it is extremely slow in biodegradation, it has found its superior applications when we use multiple component systems to formulate each component effectively [4]. Many researchers recently started investigating an effective nanoformulation system using a green phyto extract and converging it with biotechnology for an improved drug delivery system [5]. Considering the safety, low cost, and no or fewer side effects, these plant extracts are very efficient for the safe delivery of drugs. Furthermore, due to many active components like phenol, polyacetylene substances, and terpenoid presence in plant extracts, it acts as an efficient capping system for any metal carriers like zinc, silver, etc., owing to its interactions [6]. The main advantage of zinc oxide (ZnO) nanoparticle over other metal particles like silver and gold is, it can stay very active under intracellular conditions. Hence, it can alter insulin resistance and further regulate chronic inflammation, lipid, carbohydrate, and protein metabolisms through enzymatic oxidative stress [7]. Insulin resistance and induced diminution of hepatic dysregulated hepatic lipogenesis is linked commonly with obesity [8]. Obesity is a multifaceted disease involving the storage of excessive fat in the body. Various reports have investigated Zn’s concentration in the human body, which is considered toxic and has offered some indifferent results due to its level of ROS generating capability. Studies suggest that Zn’s concentration has a direct influence on the lipid profile and a high impact on metabolic syndromes. Interestingly, Zn has a close association with adipose tissue. It has significant importance in reducing triglyceride; hence Zn metal aids in an ample supplement for hepatocyte activity and increases the liver’s lipid metabolism [9]. Additionally, this helps in weight reduction and TG level without any significant change in the glucose level and lipid profile [10]. Studies suggest that the early stages of adipocyte differentiation stimulates adipogenesis through multiple upregulation pathways induced due to the presence of Zn [11]. Considering these advantages of Zn metal, we prepared nano ZnO by integrating them with the traditional medicinal plant *Dendropanax morbifera* (DM) as a natural capping agent for enriched efficacy and size [12]. Considering the applications of nano ZnOs as food supplements, nano ZnO, and a food additive, the US Food and Drug Administration (FDA) has declared ZnO as GRAS (generally recognized as safe). The primary advantages of nano ZnO is its safety with low production cost and having a wide range of biological functions as therapeutics and diagnosis to imaging [13]. Treatment with dissolved nano ZnO causes a rise in ROS concentration in the within the cells causing oxidative stress and leads to lipid neutralization [14]. Using these versatile components, we investigated the ancient *Dendropanax morbifera* (DM) for ZnO nanoparticle preparation by coloading it with tryptophan using the O/W nanoemulsion method.

*Dendropanax* belongs to the family Araliaceae, which contains around ninety-two different species found mostly on the South Korean islands. The term Dendro denotes “tree” and Panax means “Panacea”. These oriental medicinal plant extracts have been reported extensively for various infectious diseases, including obesity, skincare, anti-inflammatory diseases, and cancer application [15]. These plant extracts are from its leaf, stem, and root and the main constituents are polyacetylene compounds [16]. These extracts also contain triterpenoids and various phenolic substances, which showed an excellent anti-inflammatory property [17]. Though their components are valuable in biological applications, still, it has been used in plenty for various formulations of nutraceutical applications [16].

Owing to DM and Zn metal’s advantages, DM-ZnO nanoparticles (NPs) and Nanoemulsion (NE) are investigated highly for dietary supplements and obesity. To enhance the efficacy of DM-ZnO NPs, an essential amino acid “Tryptophan” is used for the biosynthesis of protein, is considered for NE preparation. Our immune system utilizes tryptophan starvation to restrict the proliferation of malignant cells and pathogens in the body [18]. Tryptophan breaks into kynurenine and catalyzes by IDO [19,20]. Earlier research have proposed that the IDO enzyme activity determination is from the kynurenine-to-tryptophan ratio (Kyn/Trp) [21]. The liver and the internal human organ secrete bile and store glycogen. In biological conditions, these glycogens help amino acids, minerals, and vitamins into their absorbable forms. Besides that, insulin also induces lipid generation from non-fat sources known as lipogenesis [22]. Investigations using Zn supplements either as salt or nanoparticles are considered effective in reducing the liver’s accumulated fat and thereby inducing peripheral insulin activity [12]. Recent in vitro studies suggest that nano ZnO prevented the accumulation of fat and lipid in them. With the versatile importance of tryptophan, non-ionic surfactant tween 80 and olive oil in an O/W nano emulsification method was used to load the antiobesity drug. The oil in water nanoemulsion is prepared using ultrasonication technique to entrap the bioactive components within the plant extract carrying metal nanoparticle. Tryptophan is loaded by trapping the material by the process of the ultrasonic cavitation method. Moreover, the outer capping part increases the solubility, increasing the targetability and bioavailability of the drug in metabolic conditions. Hence, considering the metabolic pathway of tryptophan, it is crucial to carry it at the lipid accumulation site to enhance its efficiency, which plays a significant role in utilizing the nano composition [23].

We prepared a biologically active nanoparticles and nanoemulsion in this study. Additionally, we carried out structural characterization utilizing the dynamic light scattering (DLS), Fourier transforms infrared analysis (FTIR), field emission-transmission electron microscopy (FE-TEM), ultraviolet–visible (UV–Vis) spectrophotometry, and FE-SEM. First, we successfully synthesized DM-ZnO-NPs from the *DM* plant extract. Next, antiobesity drug tryptophan was then loaded using food grade olive oil in a water nanoemulsion system by adding ZnO NPs water solution along with the surfactant tween 80. Further, we investigated the effect of the synthesized nanoformulation using the RAW 264.7 and 3T3-L1 cell lines. Additionally, we checked the antiobesity property with the differentiation of preadipocytes from observing the reduction of lipid droplets in the cells over a period, which implies an acceptable level of nano ZnO concentration can ameliorate the physiological homeostasis for obesity and its associated metabolic disorders.

## 2. Materials and Methods

### 2.1. Plant Materials

The *Dendropanax morbifera* sample was obtained from the southern part of Wando-gun city in South Korea from a 100 years old tree on September 2020. Around 5 kg of the plant were obtained, dried, and extracted.

### 2.2. Chemicals

Zinc nitrate hexahydrate (>98.0%), Tryptophan (Sigma Aldrich, St. Louis, MS. USA), and sodium hydroxide (>98.0%). All the media were supplied from Difco, MB Cell (Republic of Korea). Absolute alcohol, olive oil, and tween 80 was obtained from Samchun Pure Chemical Co. Ltd. (Gyeonggi-do, Korea). 3T3-L1 and RAW 264.7 Cell lines were acquired from the Korean cell line bank (Seoul, South Korea) for this study. RPMI 1640 culture medium was purchased from GenDEPOT Inc. (Barker, TX, USA). Additionally, Dulbecco’s modified eagle’s medium (DMEM) (Gibson-BRL, Grand Island, NY, USA) with 10% fetal bovine serum (FBS) and 1% penicillin/streptomycin (p/s) (WElGENE Inc., Daegu, Korea) were used for the cell experiments.

### 2.3. DM Extract Preparation

The collected sample was washed in order to remove all unwanted remains of fine particles. The washed and air-dried plant (10 g) was taken in a conical flask with 100 mL DW and extracted twice for 60 min at 100 °C under reduced pressure. To remove any suspends, the aqueous extract was separated using filtration and then centrifuged at 8000 rpm for 10 min. The clear light brown supernatant was preserved at low temperature (4–5 °C) for further experiments [16].

### 2.4. DM-ZnO NPs Synthesis from DM Extract

The *DM-*ZnO NPs were carefully synthesized employing the previously existing procedure with minor modifications during the precipitation conditions [16]. The plant extract was treated with zinc nitrate hexahydrate and sodium hydroxide as a neutralizing precursor. The synthetic conditions include the coprecipitation of nano Zn, and the further isolated nanoparticle was purified by repeated washings with water. This purification ensures the complete removal of unreacted zinc nitrate salt and other foreign substances from the extract. Of 10% *DM* extract (*w*/*v*) 200 mL was combined with 800 mL of distilled water while stirring, and then with continuous stirring (500 rpm), 0.1 mM (100 mL) of zinc nitrate salt was added to the light brown solution. To the warm solution, around 70 °C, 0.2 M (150 mL) aqueous solution of NaOH was added drop by drop with a constant stirring for over 2.0 h. During this process, the formation of precipitation begins, then thickens, and finally forms a consistent homogeneous mixture. The concoction was left to cool down by not stirring. Once the mixture was cooled down, it was centrifuged at 8000 rpm for 15 min to eliminate the unreacted plant extracts and Zn nitrate. Then, at 5 °C, a cold-water wash was carried out. The centrifuged isolated precipitate was further dried at 60 °C for around 12 h in a hot air oven. During drying the isolated Zn (OH)_2_ converts to nano ZnO as off-white powder.

### 2.5. Synthesis of DM-ZnO-Try NE from DM-ZnO NPs

**Figure 1:**
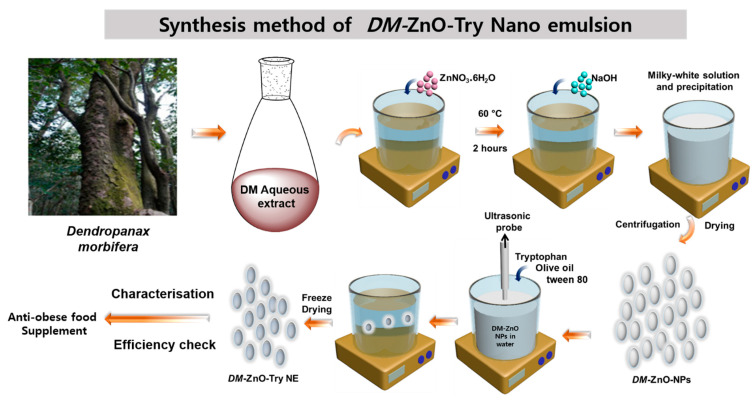
Preparation of DM-ZnO-tryptophan, oil in water nanoemulsion using the ultrasonication method.

The nanoemulsion was prepared using the ultrasonication technique with the oil in water emulsion method. The high energy was gained using the probe sonicator with an amplitude of 60% for 5 min having a 5 s pulse rate. Tryptophan was dissolved in 10% water in ethanol, along olive oil and tween 80, which is used as a surfactant for nanoemulsion preparation. The conditions for sonication were determined referring the earlier optimized conditions. Loading of tryptophan was carried out at 0–5° to avoid any excessive heat generation preferential due to the cavitation phenomenon. The stability is determined by the size for longer storage and it also helps in the drug-loading capability during the preparation of nanoformulation. Different formulation samples S1, S2, and S3 were prepared with three different conditions (Table 1). All three samples were carefully investigated for visual stability and the most stable compound is further characterized and tested for its potential bio-efficacy. Figure 1. Shows the synthetic illustration of the process.

### 2.6. Characterization

Physiochemical characterization was carried out using different analytical techniques such as size morphology, stability for the prepared nanoparticle, and nanoemulsion.

DM-ZnO NP and DM-ZnO Try NE synthesized nanoformulations were dispersed in DM water at 1 mg/mL concentration. Then, it was observed by UV–Vis spectrophotometer (Ultrospec TM-2100 pro, Biochrom Ltd, Cambridge, UK) between 200 and 700 nm for the confirmation of the composition and any foreign matters or side products during the preparation. The outer core surface was formed by the plant extract for both the NPs and NE and was confirmed by FTIR analysis (PerkinElmer Inc., Waltham, MA, USA) between 4000 and 600 cm^1^. The spectral graph was designated as transmittance (%) against wavenumber (cm^−1^). The size, surface charge, and stability of the prepared samples were characterized using DLS at 25 °C at 1 mg/mL concentration utilizing the zeta analyzer and ELSZ-2000 series (Otsuka Electronics Photal, Osaka, Japan) at pH 7.4. Further confirmation on the morphology of the prepared DM-ZnO NP and DM-ZnO Try NE was inspected using a multifunctional 200 kV-operated JEM-2100 F (JEOL, Akishima, Japan). For this analysis, a copper grid was employed to determine the samples. Topographical and elemental confirmations were investigated using FE-SEM with the following conditions and instruments. (LEO SUPRA 55, GENESIS 2000 (Carl Zeiss, EDAX, Oberkochen, Germany); gun: thermal field emission type; resolution: 1.0 nm @15 kV, 1.7 nm @1 kV, and 4.0 nm @0.1 kV; magnification: 12–900,000×).

### 2.7. In Vitro Cell Culture

#### 2.7.1. Cell Cytotoxicity

The cytotoxicity of DM-ZnO-Try against 3T3-L1 cells and RAW 264.7 was assessed using the MTT assay. In short, 3T3-L1 preadipocytes were cultured at a density of 2 × 10^3^ cells/well and incubated overnight until the cells attain full confluence. After incubating for overnight, the cells were then treated with four samples, namely DM extract, DM-ZnO NPs, DM-ZnO-Try NE, and Try at different concentrations (3.125 μg/mL, 6.25 μg/mL, 12.5 μg/mL, and 25 μg/mL) for 3 h and 24 h. At the indicated time intervals, 20 μL of MTT solution was added, and incubated at dark for 2–4 h. Similarly, RAW 264.7 cells were plated at a density of 1 × 10^6^ cells/well in a 96-well plate and incubated overnight until the cells grow to full confluence. After incubating overnight, cells were then treated with four samples, namely DM extract, DM-ZnO NPs, DM-ZnO-Try NE, and Try at different concentrations (3.125 μg/mL, 6.25 μg/mL, 12.5 μg/mL, and 25 μg/mL) for the time period of 3 h and 24 h. At the designated time intervals, 20 μL of MTT solution was added and incubated at dark for 2–4 h. The formazan crystals in each well of both cell lines were dissolved in 100 μL of DMSO, and the absorbance was measured with an ELISA plated reader at 570 nm.

#### 2.7.2. Trypan Blue Cell Viability

To detect cell viability, direct trypan blue exclusion method was used as proposed by Selcen et al., 2017 [24]. Cells were treated in a 6-well plate with DM-ZnO-Try NE samples for 3 h and 24 h at the concentrations 10 μg/mL and 12 μg/mL. Cells were then incubated with 0.2% trypan blue solution for 10 min at 20° followed by the fixation step. Further, cells were washed with PBS again after trypan blue treatment and fixed with 4% paraformaldehyde (PFA), and incubated for ½ hours at 20°. Then the PFA was removed and washed each well with 1× sterile PBS 3 times. Further, the stained and fixed cells were visualized using a 20× objective of Nikon eclipse TS100.

#### 2.7.3. 3T3-L1 Differentiation and Oil Red O Staining

3T3-L1 fibroblast preadipocytes were cultured in DMEM, which contains 10% FBS and 1% P/S, determined as complete media, in a CO_2_ incubator at 37 °C. For stimulation of adipocyte differentiation, cells were seeded at a density of 0.8 × 10^5^ per well into 6-well plates. Two days after cells got confluence (defined as day 0), they were exposed to the differentiation medium containing 0.5 mM 3-isobutyl-1-methylxanthine, 1 μM dexamethasone, and 10 μg/mL insulin. After the first three days (day 3), the differentiation medium consisting of insulin was treated for two days, and then the medium was changed every two days with the same media until the cells are fully differentiated, with or without DM-ZnO-Try NE (10 mg/mL concentration). On day 13, the 3T3-L1 preadipocytes were differentiated into mature adipocytes. The effect of these treatments on lipid accumulation by adipocytes was examined by the Oil Red O (ORO) staining method as follows. In brief, dissolved 0.7 g Oil Red O powder in 200 mL of 100% isopropanol and stirred overnight. The solution was filtered through a 0.22 μm membrane filter and stored at 4 °C. Fresh Oil Red O working solutions were prepared by mixing the stock solution with distilled water (6:4) and then incubated for 20 min following filtration. The cells were washed three times with PBS and fixed with 10% formaldehyde in PBS at 25 °C for 1 h. Once the cells were fixed, they were washed with distilled water thrice and then stained the differentiated cells with Oil Red O working solution at 25 °C for 2 h. Finally, the cells were rinsed again three times using distilled water and photographed with an Nikon instruments, Melville, NJ, USA. After taking pictures, to check the intracellular lipid content, the Oil Red O dye was eluted with isopropanol and was measured with an Epoch^®^ micro volume spectrophotometer at 520 nm.

## 3. Results

### 3.1. Physicochemical Properties of the Synthesized Nanoformulation

Nano composition was synthesized using different concentrations of the nanoformulation method. Different concentrations with S1, S2, and S3 were prepared and explained in Table 1. The sample composition was detailed in Table 1 along with water and oil (olive oil). The nanoformulation system of W/O or O/W is determined by the composition of the surfactant, water, and oil. Around 50–90% of the water with 1.5–10% of the surfactant is considerate for oil in the water method. The higher the concentration of the surfactant, the higher the toxicity of the material so we considered the safe proportion of 5% *w*/*w* for our composition. The nanoformulation system was determined by W/O or O/W based on the composition of the olive oil, water, and surfactant. A recently reported composition of 90% water around 10% of the surfactant was found to be suitable for this stable formulation [25]. Initial visual observation was made for the coagulation behavior of the three different concentration nanoparticles. After a day, sample 1 and sample 3 turned slowly towards milky and translucent, whereas sample S2 showed stability for more than 2 days at 25 °C and 40 °C. Further, to check the precipitation ability of S2, it was centrifuged at 3000 rpm for 30 min and no precipitation was observed. Based on the initial screening, we further proceeded with the preparation of nanoemulsion using tryptophan for our preparation. For understanding the stability of the prepared nanoparticle and nanoemulsion, size correlation measurements were performed over 10 days using DLS measurements. The stability difference is mainly due to the nature of the materials, interactions due to the binding materials, and the composition of the oil, water, and surfactants. The DLS data suggests that the formulation of nanoemulsion is homogenetic with a low polydispersity value for excellent stable formulation. For further experiments, S2 was chosen based on the preliminary results. DM-ZnO-Try NE contains 75% aqueous part containing 75.0 mg of DM-ZnO NPs and olive oil (8%) and a surfactant (7%) and was also loaded with 5% tryptophan (25.0 mg).

### 3.2. UV–Vis Analysis

**Figure 2:**
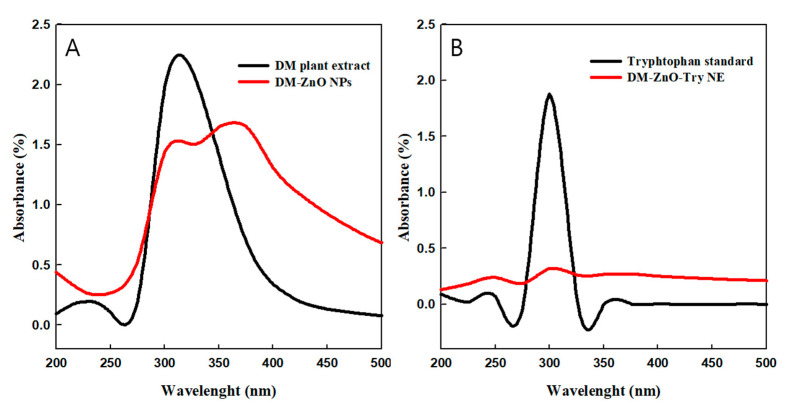
UV–Visible spectra of (**A**) DM-ZnO nanoparticles (NPs) with DM extract and (**B**) DM-ZnO-Try NE with standard tryptophan.

UV absorbance spectra of the plant extract, DM capped nano ZnO [16], drug-loaded DM-ZnO nanoemulsion, and standard tryptophan was measured and represented as Figure 2A,B. The UV spectra confirm the UV absorbance data, which indicates the strong sharp surface at the plasmon resonance wavelength (λ_spr_) at 350 confirming the successful synthesis of DM-ZnO NPs. Strong signals at 320 suggest the presence of polyphenols and anthocyanins in the extracts. These polyphenols and anthocyanins are responsible for interactions in the formation of nanoparticles. Both NPs and NE showed a characteristic signal at 250 nm, which validates the capping of *DM* extract in the formulation (Figure 2A). Additionally, both NPs and NE validate the presence of tryptophan with evidence of a peak at 300 nm and confirming the entrapment of Try during the loading process of nanoemulsion preparation (Figure 2B). For further confirmation, the structural formation changes by SEM and TEM characterizations along with elemental mapping for the presence of metal ion presence were observed.

### 3.3. FT-IR Spectroscopic Analysis

**Figure 3:**
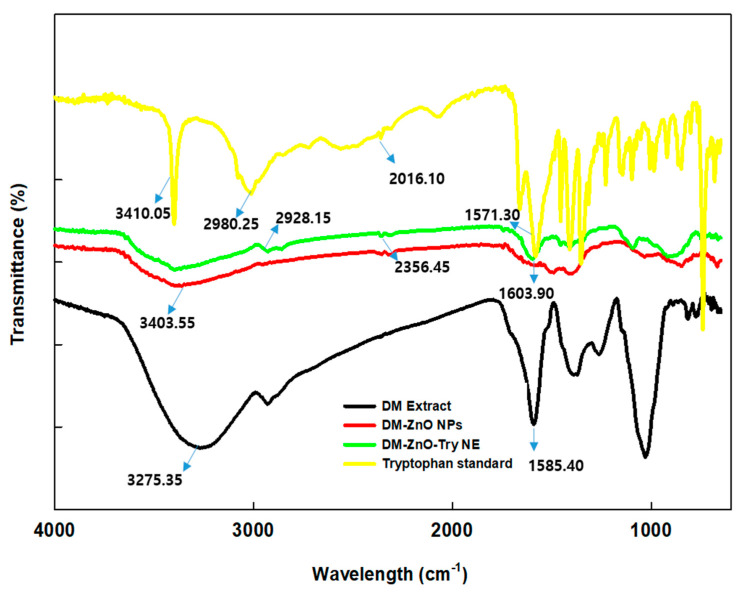
FT IR spectra of tryptophan standard (yellow), DM-ZnO NPs (Red), DM-ZnO-Try NE (Green), and DM plant extract (Black).

The formation of nanoemulsion and nanoparticles require slightly effective conditions for better preparation. To understand the nature, stability, and formulation of the nanoemulsion, FT-IR was carried out for the characteristic changes. FT-IR spectra of DM plant, DM-ZnO NPs, and DM-ZnO-Try NE along with standard tryptophan were performed and represented in Figure 3. Both NPs and NE showed the characteristic peaks for functional groups of DMs, which had phenolic (-OH group) at 3400.0 cm^−1^ and secondary amine (-NH) along with the absorbance at 2850 cm^−1^ and 2350 cm^−1^ corresponding to –CH stretch and –C=O stretching. Stretching vibration -C=C was confirmed by the presence of signals at 1500 cm^−1^. Both NP and NE exhibit the characteristic absorbance peaks concerning plant extract. Sharp peaks for tryptophan and nanoemulsion were compared for the characteristic peaks and these observations further confirmed the successful completion [26].

### 3.4. Size Measurement Analysis and Surface Charge

**Figure 4:**
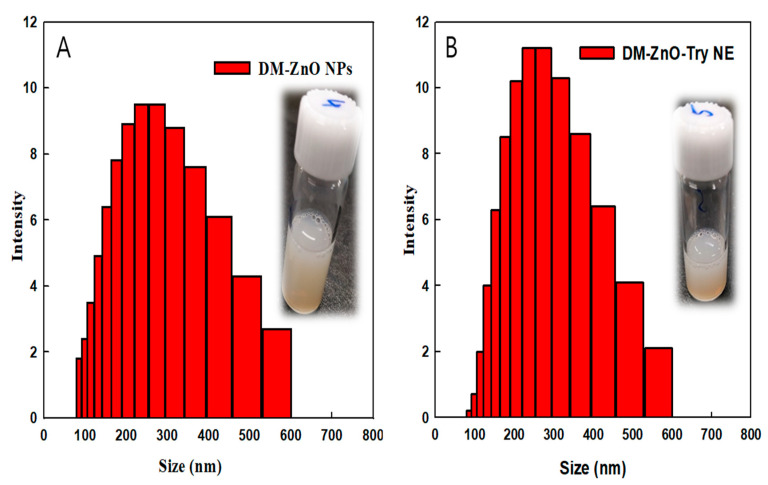
Size distribution analysis using the dynamic light scattering (DLS) analyzer for (**A**) DM-ZnO NPs and (**B**) DM-ZnO-Try NE.

The hydrodynamic size of the prepared nanoparticles and nanoemulsion were determined using DLS analysis using 1 mg/mL concentration in water (pH7.4) at around 25 °C. DM-ZnO NPs (Figure 4A) possess about 146.26 ± 3.3 nm with a PDI value of about 0.5 and the tryptophan entrapped using the oil and water method nanoemulsion product of DM-ZnO-Try NE (Figure 4B) possesses about 151.16 ± 3.6 nm with the PDI value of about 0.13 due to the coloading of the new moiety. The surface charges of both nanoparticles and nanoemulsion products are determined using a Zeta analyzer. DM-ZnO NPs and DM-ZnO-Try NE were confirmed as −13.06 ± 0.3 mV, which confirms the effective loading and the stability of the nanoemulsion product prepared and shows no significant changes in the zeta potentials were observed. The negative surface is due to the presence of various polyphenols from the OH−, COO−, and CO− of DM extract. This negative charge ZnO NPs associated with aiding the dispersity of the particles and prevents the surface attachments and thereby stabilizes the nanoparticles without any aggregation due to their electrostatic repulsions [27]. The DLS histogram confirms the dispersion of the prepared nano products, which was confirmed further by other physiochemical characterization techniques of FESEM and FETEM.

### 3.5. Size Stability

**Figure 5:**
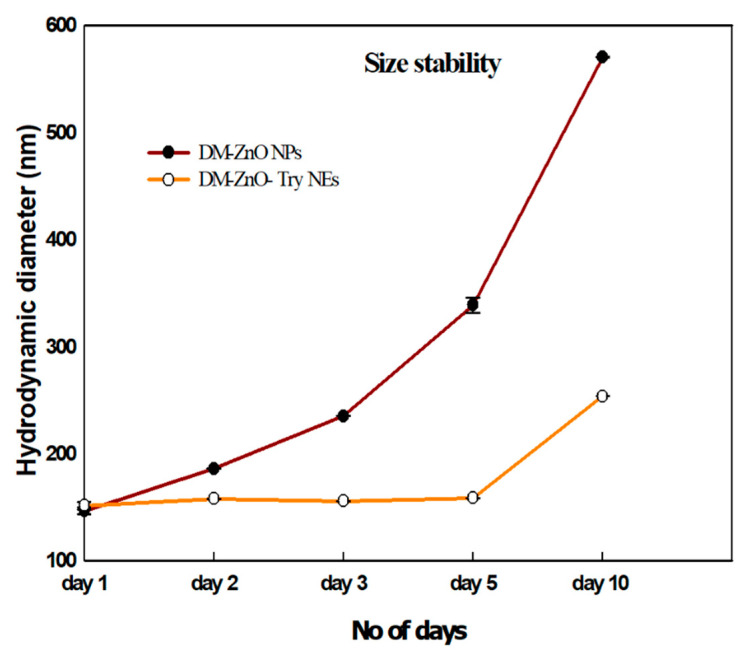
Size stability of DM-ZnO NPs and DM-ZnO-Try NE with the time factor.

To observe the stability of the prepared nanoparticles and nanoemulsion, they were dispersed in DIW and their size distribution in Figure 5 was measured as a function of time. Based on the particle size DM-ZnO-Try NE was found to be stable up to 5 days, whereas the NPs slightly started to swell and found an increase in the particle size due to the difference in amphiphilic nature of the material. However, the size is constant up to day 5 for prepared nanoemulsion, which is found to have higher stability due to the loading of tryptophan product and its binding nature. This confirms its safety and stability for longer circulation in physiological conditions.

### 3.6. FE-TEM, Elemental Mapping, and FE-SEM Analysis

**Figure 6:**
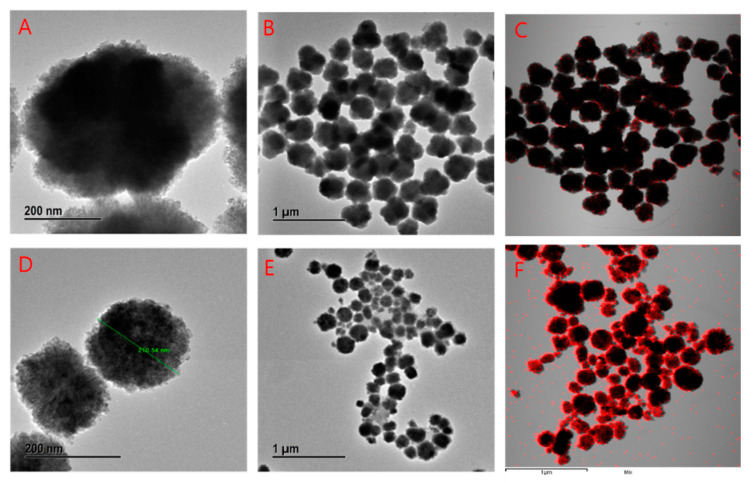
FE-TEM analysis of DM-ZnO nanoparticles, (**A**) multiple images at 200 nm bar range (no aggregation), (**B**) multiple images at 1µm bar range (no aggregation), (**C**) elemental mapping and DM-ZnO NPs, (**D**) multiple images for DM-ZnO-Try-NE at 200 nm range, (**E**) multiple images at 1 µm bar range (no aggregation), and (**F**) elemental mapping and of DM-ZnO-Try nanoemulsion.

The morphology of the nanoparticles and nanoemulsions were characterized further using FE TEM. The unimodal size distribution with an average diameter of around 200 nm was observed. The spheroidal structure of nanoparticles for nanoparticles and nanoflowers with the combination of 3–4 board petal-like structures for nanoemulsion were noticed. Figure 6B,E, shows at the 1 μm range that did not aggregate with each other. The same trend is observed for the nanoemulsion product. Noticeable change of petal shape is due to the entrapment of tryptophan. Furthermore, this petal-like structure can damage the cell wall and cell mechanisms greater than its spherical shape. Elemental mapping revealed the distribution of Zn (red dot) Figure 6C,F for both nanoparticles and nanoemulsion. 

**Figure 7:**
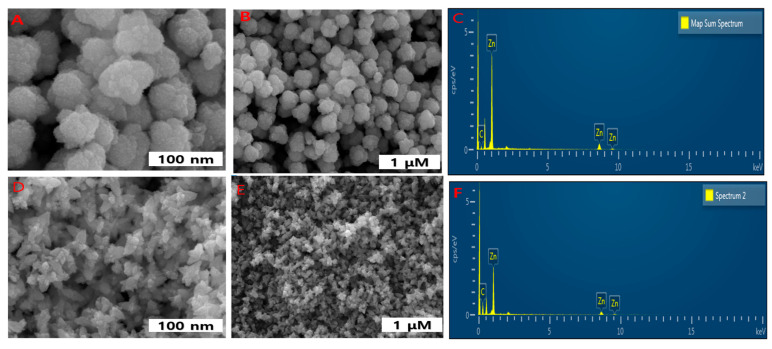
FE-SEM analysis of DM-ZnO nanoparticles exhibited spheroid shape in (**A**,**B**) and DM-ZnO-Try nanoemulsion exhibited flower shape in (**D**,**E**). EDS analysis for DM-ZnO-NPs (**C**) and DM-ZnO-Try-NE (**F**).

For further confirmation of the morphological understanding, it was subjected to SEM. The morphological image and chemical composition was determined by EDX Figure 7, which coincides with TEM images. Interestingly, the biggest amounts of ZnO NPs were similar in dimension with a small number of large particles. The ZnO NPs and NEs also showed small agglomeration, which is typical during the green synthesis of NPs. This agglomeration is attributed to the fact that green-synthesized NPs possess a higher surface area and they strongly stick to each other with considerable affinities to form asymmetrical clusters Figure 7A,B for DM-ZnO NPs and Figure 7D,E for DM-ZnO-Try NE. The EDX spectrum shows strong major peaks, which confirms the elemental distribution of the ZnO NPs and ZnO NE products Figure 7C,F.

### 3.7. In Vitro Cell Cytotoxicity Analysis

#### 3.7.1. Effects of DM-ZnO-Try on 3T3-L1 and RAW 264.7 Cell Viability

**Figure 8:**
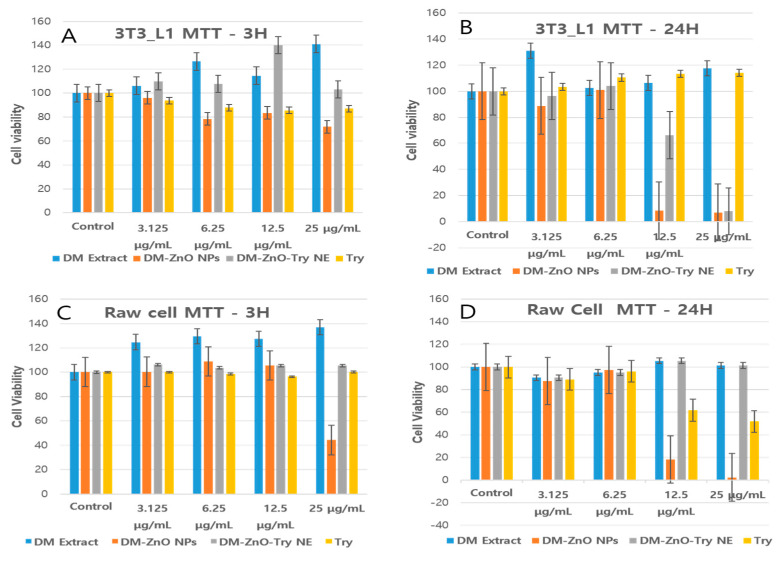
In vitro cytotoxicity analysis for DM extract, DM-ZnO-NPs and DM-ZnO-Try-NE, and Try in 3T3-L1 cell line (**A**) 3 h and (**B**) 24 h and raw cell line (**C**) 3 h and (**D**) 24 h. Each value is expressed as the mean ± standard error of three independent experiments. *p* < 0.001 compared with control.

**Figure 9:**
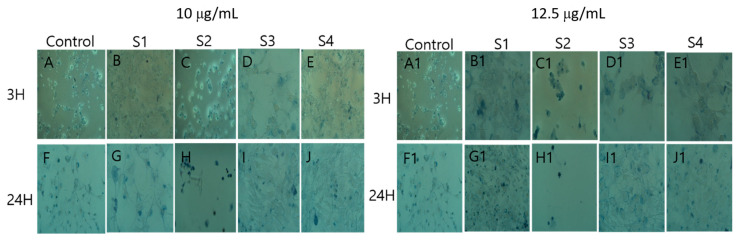
Trypan blue cell viability images for control, (S1) DM extract, (S2) DM-ZnO-NPs, (S3) DM-ZnO-Try–NE and (S4) Tryptophan at 10 µg/mL (**A**–**J**) and 12.5 µg/mL (**A1**–**J1**) for 3 h and 24 h time point at 20× magnification.

The effects of DM-ZnO-Try NE on cytotoxicity of 3T3-L1 preadipocyte and RAW 264.7 cells are shown in Figure 8. To check for potential toxicity of DM-ZnO-Try NE on 3T3-L1 and RAW 264.7 cells, viability after exposure to a series of concentrations, and for two (3 h and 24 h) function of time, was determined using the MTT(3-(4,5-dimethylthiazol-2-yl)-2,5-diphenyltetrazolium bromide) assay. The various concentrations (0, 3.125, 6.25, 12.5, and 25 mg/mL) of DM-ZnO-Try NE did not affect cell viability of the RAW 264.7 cells compared with the control till 12.5 mg/mL as demonstrated in Figure 8. As presented in Figure 8, 3T3-L1 cells treated with various concentrations of different samples (DM extract, DM-ZnO NPs, DM-ZnO-Try NE, and Try) showed that the tested compound was not remarkably toxic until 6.25 μg/mL to 12 μg/mL of DM-ZnO-Try NE, where more than 80% of cells were viable. Therefore, further, we used 10 mg/mL (concentration between 3.125 and 12 mg/mL or the nearest concentration to 12 mg/mL) and 12 mg/mL of DM-ZnO-Try NE for the trypan blue toxicity assay. The trypan blue assay was shown in Figure 9A,B to be less toxicity at this selected concentration and visual live-cell morphology behavior aids to perform cell differentiation measurements and determine the intracellular lipid accumulation Hence, this concentration (10 mg/mL) was chosen as the maximum and used for further studies.

#### 3.7.2. Effect of DM-ZnO-Try on Intracellular Lipid Accumulation in 3T3-L1 Cells

**Figure 10:**
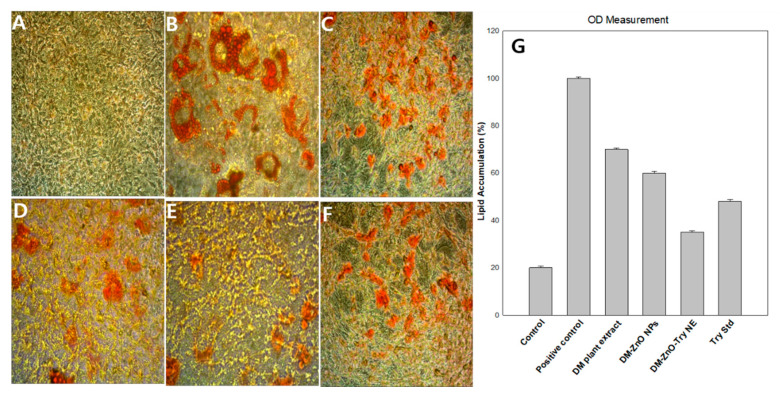
Cell differentiation of (**A**) control, (**B**) positive control, (**C**) DM extract, (**D**) DM-ZnO-NPs, (**E**) DM-ZnO-Try–NE, and (**F**) tryptophan at 10 µg/mL and at 20× magnification (**G**) OD measurements for lipid accumulation.

To test whether DM-ZnO-Try NE inhibits intracellular triglyceride accumulation, complete media containing insulin, dexamethasone, and IBMX was used to induce 3T3-L1 preadipocyte differentiation in the presence or absence of 10 mg/mL different samples in Figure 10A–F (control, positive control, DM extract, DM-ZnO NPs, DM-ZnO-Try NE, and Try, respectively). On day 13, excess lipid accumulation was observed inside the differentiated positive control cells. The DM-ZnO-Try NE treated cells had lesser lipid droplets compared to the positive control. Lipid accumulation and the occurrence of the adipocyte phenotype of the matured adipocyte cells were then assessed by staining with Oil Red O. The microscopic observation of the Oil Red O stained cells shows in Figure 10G that the lipid accumulation was notably decreased by DM-ZnO-Try NE samples. The results were quantified by measuring the absorbance of the solubilized stained lipid droplets at 520 nm. The results showed that the treatment with different samples (DM extract, DM-ZnO NPs, DM-ZnO-Try NE, and Try) at 10 mg/mL and DM-ZnO-Try NE resulted in notable reductions of lipid accumulation in the cells in Figure 10E than the other samples due to its synergetic effect. The lipid content was decreased by 70%, 60%, 35%, and 48% at the concentration 10 mg/mL of the DM extract, DM-ZnO NPs, DM-ZnO-Try NE, and Try, respectively as shown in Figure 10G. Adipogenesis is the stage where the preadipocytes mature into adipocytes in the process of cellular differentiation. It is accompanied by lipid accumulation and changes in various adipogenesis related gene expression [28]. These results indicate that DM-ZnO-Try NE has antiadipogenic properties and can be used as an effective antiobesity drug due to its inhibitory effect on adipocyte differentiation.

## 4. Conclusions

*Dendropanax morbifera,* an ancient medicinal plant native to South Korea, its 100-year-old plant extract is considered for its therapeutic applications to date. Our investigation represents the understandable approach of green synthesis for functional food nanoformulation. A natural coating surface using an ancient DM plant extract mediated with ZnO nanoparticles carrying an antiobese drug tryptophan is formulated. The process of preparation involves the O/W nanoemulsion method using high energy ultrasonication with non-ionic surfactant interactions, to enhance the trapping and efficiency of active ingredients at the targeted site. The synthesized nanoformulation was confirmed by some critical physicochemical characterization using DLS, UV, FT-IR, FE-TEM, and FE-SEM, and its stability was discussed for a longer circulation of over five days. In vitro behavioral changes of the nanoformulation initiate at the targeted site, where it undergoes the process of degrading its outer capping layer to further expose the Zn^2+^ ion and tryptophan. Due to this exposure of Zn^2+^ ion, it affects the mitochondrial damages due to its transition permeability and induces oxidative stress to reduce fat depositions and thereby, it increases the bioactivity of tryptophan causing dual effects by reducing the accumulation of lipids at the fat deposited site. This phenomenon is investigated with 3T3-L1 preadipocytes and RAW 264.7 cells at 10 μg/mL concentration. DM-ZnO-Try NE has shown lower toxicity in RAW 264.7 and 3T3-L1 preadipocytes and has shown an excellent efficiency in 3T3-L1 preadipocytes by reducing around 45% of the lipid droplets. This increased activity of the nanoemulsion may be due to its particle size and morphology. Oil Red O staining assay was performed to confirm the lipid reduction, which was then further verified using the OD measurements. This evidence signifies that the DM-ZnO-Try NE substantially reduces more lipid droplets in comparison with its counterpart of DM-ZnO NPs and standard tryptophan. Hence, it could be considered in the treatment of obesity and to study other related metabolic disorders such as heart disease, stroke, and type 2 diabetes by investigating in detail. This study suggests that the enhanced efficacy of the safe formulation can be used as a functional food for commercial applications, due to its low cost and biodegradable natural sources.

## Figures and Tables

**Table 1:** Conditions for nanoemulsion preparation.

Sample (Name)	DM-ZnO NPs (Extract) (mg)	Olive Oil (%)	Tryptophan Drug (mg)	Surfactant (Tween 80) (%)
S1	75.0	10	25.0	3
S2	75.0	8	25.0	7
S3	75.0	5	25.0	10

## Data Availability

We wish not to share the data for publically available, as further research progress is going on with this basic research.
